# The phonological and visual basis of developmental dyslexia in Brazilian Portuguese reading children

**DOI:** 10.3389/fpsyg.2014.01169

**Published:** 2014-10-14

**Authors:** Giseli D. Germano, Caroline Reilhac, Simone A. Capellini, Sylviane Valdois

**Affiliations:** ^1^Investigation Learning Disabilities Laboratory, Department of Speech and Hearing Sciences, São Paulo State UniversityMarília, Brazil; ^2^Department of Cognitive Science, Johns Hopkins UniversityBaltimore, MD, USA; ^3^Centre National de la Recherche Scientifique, UMR 5105Grenoble, France; ^4^Laboratoire de Psychologie and Neurocognition, Université Grenoble AlpesGrenoble, France

**Keywords:** developmental dyslexia, phonological processing, visual processing, subtypes, visual attention span

## Abstract

Evidence from opaque languages suggests that visual attention processing abilities in addition to phonological skills may act as cognitive underpinnings of developmental dyslexia. We explored the role of these two cognitive abilities on reading fluency in Brazilian Portuguese, a more transparent orthography than French or English. Sixty-six children with developmental dyslexia and normal Brazilian Portuguese children participated. They were administered three tasks of phonological skills (phoneme identification, phoneme, and syllable blending) and three visual tasks (a letter global report task and two non-verbal tasks of visual closure and visual constancy). Results show that Brazilian Portuguese children with developmental dyslexia are impaired not only in phonological processing but further in visual processing. The phonological and visual processing abilities significantly and independently contribute to reading fluency in the whole population. Last, different cognitively homogeneous subtypes can be identified in the Brazilian Portuguese population of children with developmental dyslexia. Two subsets of children with developmental dyslexia were identified as having a single cognitive disorder, phonological or visual; another group exhibited a double deficit and a few children showed no visual or phonological disorder. Thus the current findings extend previous data from more opaque orthographies as French and English, in showing the importance of investigating visual processing skills in addition to phonological skills in children with developmental dyslexia whatever their language orthography transparency.

## INTRODUCTION

Developmental dyslexia has been reported in different languages but only a few studies have been done in Portuguese reading children, and most concerned European Portuguese. The present study explored the cognitive underpinning of developmental dyslexia in Brazilian Portuguese, a language that differ substantially from European Portuguese. The children with developmental dyslexia participants’ phonological skills were assessed assuming that phonological awareness contributes to normal reading acquisition and is typically found impaired in children with developmental dyslexia, whatever their alphabetic language (Sprenger-Charolles et al., 2000). The children’s visual and visual attention (VA) skills were further investigated. Much evidence indeed suggests that some children with developmental dyslexia show a VA span disorder – namely a difficulty to simultaneously process multiple elements – that contributes to their poor reading performance independently of their phonological skills. Evidence was provided for French and English speaking children with developmental dyslexia (Bosse et al., 2007) and in the recent case study of a bilingual French–Spanish dyslexic girl (Valdois et al., 2014), suggesting that the disorder might extend to all kind of orthographies regardless of their transparency. The current study will provide support to this hypothesis in showing that the visual attentional skills of Brazilian Portuguese children with developmental dyslexia significantly and independently account for their poor reading performance. Last, we will search for dyslexia subtypes characterized by distinct cognitive disorders. We will more specifically explore whether cognitively distinct subgroups characterized by a single phonological disorder or a single VA disorder do exist in the Brazilian population as previously described in French and English speaking children.

### DEVELOPMENTAL DYSLEXIA IN PORTUGUESE

The manifestations of developmental dyslexia differ from one language to the other. Indeed, the ease of reading acquisition varies between languages because of differences in language transparency. Language transparency refers to the degree of consistency of mappings between orthographic and phonological units. The most transparent languages rely on consistent one-to-one mappings between letters and phonemes while the opacity (or depth) of languages increases with the number of orthographic inconsistencies and irregularities. Seymour et al. (2003) proposed a classification of the European languages according to their orthographic transparency/depth. Portuguese was considered as more consistent than French or English but deeper than Italian, Spanish, or Dutch. In line with this view, typically developing Portuguese-speaking European children show a reading accuracy level of performance comparable to that of children with deeper orthographies but Portuguese word or pseudo-word reading is typically faster (Seymour et al., 2003). Only a few studies have been conducted in Brazilian Portuguese. Brazilian Portuguese differs significantly from European Portuguese. Letters that are mute in the European dialect are typically omitted in Brazilian Portuguese and variant diacritics are used to disambiguate alternative pronunciations. It follows that Brazilian Portuguese is of greater transparency than European Portuguese, at least for reading (Cardoso-Martins, 2006). Thus, most words can be read successfully through phonological decoding, and even reading fluency can partly reflect the ability of the reader to apply grapheme–phoneme correspondences (Pinheiro et al., 2008). In Brazilian Portuguese, phonological reading predominates from beginning readers to the third grade but with age, performance gradually relies on lexical knowledge and sight word vocabulary (Oliveira and Capellini, 2010; Mota et al., 2012). Reliance on lexical knowledge is required as some graphemes may represent different phonemes and some correspondences are very rare (as “x” that can be pronounced as /∫/ or /z/) and only used in some “irregular words.” Thus, as reported in most alphabetic languages whatever their transparency (Davies et al., 2013), Brazilian Portuguese older children typically read words faster and more accurately than pseudo-words, suggesting they rely on two analytic and global (or lexical) reading procedures (Pinheiro, 1995).

Orthographic transparency is known to influence the rate of reading acquisition (Seymour et al., 2003; Ziegler et al., 2010) and thus affects the degree of reading difficulties. Readers with developmental dyslexia from deep orthographies suffer much severe reading disorders than children from transparent orthographies (Landerl et al., 1997; Bergmann and Wimmer, 2008). Accordingly, poor reading accuracy and slow reading speed are typically reported as defining features of developmental dyslexia in deep orthographies (like French or English) while slow reading speed is the key feature in transparent orthographies (like Spanish or Italian). Only a few studies have explored the manifestations of developmental dyslexia in Portuguese. Sucena et al. (2009) reported that European-Portuguese children with developmental dyslexia exhibited reading disorders on irregular words and pseudo-words, relative to chronological age matched typical readers. They performed worse than controls in terms of both accuracy and fluency, as typically reported in deep orthographies. In line with these findings, available data in Brazilian Portuguese suggests that both reading speed and reading accuracy are impaired in developmental dyslexia (Ávila et al., 2009). In the current study, participants with developmental dyslexia will be recruited based on their word and pseudo-word reading accuracy but reading fluency will be taken as a more sensitive experimental measure of their reading abilities. Accordingly, we will focus on the cognitive mechanisms that better account for reading fluency in Brazilian Portuguese children, with or without developmental dyslexia.

### PHONOLOGICAL PROCESSING, READING ACQUISITION, AND DEVELOPMENTAL DYSLEXIA

Phonological awareness is closely associated with the process of reading acquisition, regardless of the language degree of consistency between orthographic and phonological mappings (Duncan et al., 2013; Moll et al., 2013). Phonological awareness refers to the ability to identify and manipulate phonological units as syllables or phonemes. The development of phonological awareness enables the child to understand the alphabetic principle and acquire the mappings between graphemes and phonemes, thus playing an important role in the development of decoding skills at the beginning of literacy acquisition. However, phonological awareness further contributes to the establishment of word specific knowledge and thus modulates reading acquisition all along primary school (Share, 1999, 2004). Accordingly, many studies showed that phonological awareness is a concurrent (Bosse and Valdois, 2009; Moll et al., 2013) and longitudinal (Muter et al., 2004; Nikolopoulos et al., 2006; Caravolas et al., 2012) predictor of reading skills, in both opaque and transparent orthographies. Vaessen et al. (2010) showed that phonological awareness substantially contributed to reading fluency in European Portuguese. However, in Portuguese as in most transparent languages than English (De Gelder and Vroomen, 1991; Bosse and Valdois, 2009), the contribution of phonological awareness to reading fluency was found stronger at the beginning of reading development, but then declined with reading expertise.

Given the importance of phonological awareness for literacy acquisition, deficits in phonological awareness have been systematically reported in developmental dyslexia whatever the orthographic depth of the language (Vellutino et al., 2004). Portuguese is not an exception to the rule. There is evidence that European Portuguese children with developmental dyslexia children exhibit a deficit in phonological skills (Sucena et al., 2009). A phonological disorder was also reported in Brazilian Portuguese poor and children with developmental dyslexia readers. Some studies made in Brazil (Capellini et al., 2008; Germano and Capellini, 2008, 2011; Germano et al., 2008, 2009) compared the phonological abilities of two groups of children with developmental dyslexia and good readers. The participants were administered phonemic and syllabic deletion, transposition and blending tasks. Results showed that children with developmental dyslexia performed poorer than the controls in all the phonological tasks whatever the unit (phoneme or syllable) to be manipulated. However, recent evidence that VA affects reading performance and may partly account for the child phonological skills (Vidyasagar and Pammer, 2010) requires investigating the unique contribution of phonological skills to reading performance after control of the child VA skills. For this purpose, our Brazilian Portuguese participants were submitted to testing protocols, all standardized for Portuguese Brazilian population, such as Metalinguistic Skills Evaluation Battery (PROHFON Battery) for the assessment of phonological skills (Germano and Capellini, 2011) but also tasks assessing their VA abilities.

### VISUAL ATTENTION AND DEVELOPMENTAL DYSLEXIA

Despite strong evidence that difficulties with phonological processing result in developmental dyslexia, it is quite obvious that all individuals with developmental dyslexia do not have a phonological disorder. Typically, phonological processing was found intact in children with a selective irregular word reading disorder but preserved pseudo-word reading (Castles and Coltheart, 1996; Valdois et al., 2003; Dubois et al., 2007, 2010). Cases of neglect dyslexia or letter position dyslexia have also been reported whose reading impairment cannot be accounted for by a phonological disorder (Friedmann and Nachman-Katz, 2004; Friedmann and Gvion, 2005). In line with the multifactorial approach (Pennington, 2006), more and more studies in the last decade revealed that abilities tapping into some kind of visual attentional processing contributed to reading acquisition and were impaired in developmental dyslexia (Boden and Giaschi, 2007; Bosse and Valdois, 2009; Franceschini et al., 2012). Difficulties in processing multiple element configurations has been well documented in developmental dyslexia (Pammer et al., 2004; Hawelka and Wimmer, 2005; Dubois et al., 2010; Lobier et al., 2012a,b). These difficulties might reflect deficits in the allocation of attention across the distinct visual elements that compose a global shape.

In line with these findings, a VA span disorder, namely a limitation in the number of distinct visual elements that can be processed simultaneously in a visual display, was found to characterize a subset of French and British children with developmental dyslexia who showed no phonological disorder (Bosse et al., 2007). The VA span is expected to play an important role in reading acquisition, as it delineates the amount of orthographic information that can be processed at each step of the reading process (Valdois et al., 2004). A large VA span that encompasses the whole word letter string allows each of its constituent letters to be accurately identified in parallel so that the word can be identified as a whole through a fast parallel reading procedure. In contrast, a deficit in visual processing capacity that limits the ability of the VA span to spread over the whole word results in slow serial decoding of the regular words and misreadings of the irregular words in opaque languages. Accordingly, reduced VA span in French and British children with developmental dyslexia readers was found to account for their poor reading outcome, independently of the child phonological skills (Bosse et al., 2007; Zoubrinetzky et al., 2014). In an opaque language as French, the VA span relates to reading accuracy for all types of items (words and pseudo-words) and shows a strong and sustained influence on irregular word reading over grades (Bosse and Valdois, 2009). A sufficiently large VA span should be essential to cope with inconsistencies and irregularities that characterize opaque orthographies as in French or English. However, the VA span impact on reading performance is not restricted to the reading by sight procedure. A large enough VA span is further required to process multi-letter sublexical units (i.e., multiletter graphemes or syllables) and can thus affect sublexical processing when impaired. Supporting evidence was provided for French and English (Bosse et al., 2007) showing that poor VA span independently contributed to poor pseudo-word reading in developmental dyslexia (see also Zoubrinetzky et al., 2014). However, the role of VA span in more transparent orthographies – as Brazilian Portuguese – that rely on smaller orthographic units has not yet been investigated.

### PURPOSE OF THE CURRENT STUDY

In this study, we will address two main issues. More and more evidence suggests that VA span abilities (i.e., multiple elements visual simultaneous processing) contribute to reading performance in opaque languages like French or English. Our first goal will be to demonstrate that the impact of VA span to reading performance extends to a more transparent language like Brazilian Portuguese. Positive evidence would reinforce the view that VA span acts as a second core disorder in developmental dyslexia, regardless of language transparency. Previous evidence from opaque languages that phonological and VA skills independently contribute to the poor reading outcome of children lead to identify different subtypes of developmental dyslexia characterized by distinct cognitive disorders. The second main issue of the current study was to provide first evidence for cognitively distinct subtypes in Brazilian Portuguese children with developmental dyslexia. Evidence for distinct subtypes seems more natural in languages as French or English that rely on two whole word and sublexical procedures and in which selective disorders of irregular word or pseudo-word reading have been reported. Evidence for distinct subtypes in transparent languages seems less intuitive but is nevertheless compatible with evidence that similar reading procedures are involved in opaque and transparent orthographies. Moreover, evidence for distinct subtypes of developmental dyslexia in Brazilian Portuguese children will be of great relevance for the diagnosis and remediation of developmental dyslexia in this language.

## MATERIALS AND METHODS

The Research Ethics Committee of the institution of origin approved this study, protocol number 182/2011.

### PARTICIPANTS

Sixty-six Brazilian Portuguese children took part in this experiment. All were monolingual native speakers of Portuguese who lived in the Sao-Paulo state (Brazil). The 33 participants of the control group were typically developing children with no history of learning disability. They were strictly matched to the participants with developmental dyslexia for chronological age by pairing in years and months each control participant with each child with developmental dyslexia (mean CA = 10 years 2 months, SD = 11,5 months; range: 8;0–11;11). The control participants had normal visual acuity and hearing and were identified by their teachers as being average or good readers. Their general cognitive abilities assessed at school using either the WISC-R or the Raven matrices were within the normal range. However, information on their exact IQ score was not provided for ethical reasons and thus, cannot be reported here for the group. All the participants of the students signed the term of free and informed consent (TFIC).

The developmental dyslexia group consisted of 33 children (24 males). The diagnosis of dyslexia was done by an interdisciplinary team of pediatric neurologists, neuropsychologists, educational psychologists and Speech Language Therapists from the Laboratory of Investigation of Learning Deviations of the Center for the Study of Education and the Faculty of Health Sciences – São Paulo State University “Júlio de Mesquita Filho” – UNESP – Marília – São Paulo – Brazil and from the Laboratory of Learning Deviations [Hospital of the Faculty of Medicine (HC/FM) – UNESP – Botucatu – São Paulo – Brazil]. The participants with developmental dyslexia were selected based on standard exclusion criteria (American Psychiatric Association, 1994). They had normal IQ (mean = 93; SD = 7.5) based on WISC-III (Wechsler, 2002) and attended school regularly. None of them had any history of neurological illness or brain damage. All had normal or corrected-to-normal vision and hearing. The children referred by the municipal secretary of Education were administered a multidisciplinary screening including tests of single word and pseudoword reading (Capellini et al., 2012a), and word and pseudoword spelling (Capellini et al., 2012b). In reading, words and pseudowords were matched in syllabic structure. The word spelling task included regular and irregular words of high and low frequency. The children were considered as developmental dyslexia when underperforming (at least 1.6 SD below the mean) in reading and spelling as compared to normative data from children of the same chronological age and educational level (Salles and Parente, 2007; Dias and Ávila, 2008; Capellini et al., 2012a). Tasks sentence and text comprehension were further administered. The sentence comprehension test was composed of 12 sentences the child had to read and then execute the corresponding order (i.e., “Draw a picture with a tree with three apples”). The text comprehension test required the child to read four small texts and answer four questions following each text.

As shown on **Table 1**, the two groups significantly differ in reading and spelling accuracy for both words and pseudo-words. The children with developmental dyslexia further show lower sentence and text comprehension than the controls.

**Table 1:** Performance of the participants, median (standard-deviation) on the screening tests of reading and spelling.

	Developmental dyslexia group *N* = 33	Control group *N* = 33	*t* value, *p* value
**Reading**	**Median (SD)**	**Median (SD)**	
Word identification (/30)	21.48 (9.07)	29.91 (0.29)	*t* = -6.11, *p* < 0.00001
Pseudo-word identification (/30)	16.61 (9.39)	29.06 (1.00)	*t* = -7.58, *p* < 0.00001
Sentence comprehension (/12)	8.15 (5.00)	11.78 (0.59)	*t* = -4,14, *p* < 0.001
Text comprehension (/16)	7.12 (5.97)	14.12 (1.49)	*t* = -6,55, *p* < 0.00001
**Spelling**
Word spelling (/30)	19.64 (6.61)	28.39 (0.56)	*t* = -7.58, *p* < 0.00001
Pseudo-word spelling (/10)	5.70 (2.27)	9.45 (0.67)	*t* = -9.12, *p* < 0.00001

### EXPERIMENTAL MATERIAL

The experimental session included a text reading task, three metaphonological tasks and three visual processing tasks. The children were assessed individually, at school in a quiet room.

#### Text reading

The text administered for the assessment of oral reading was the “Umbrella,” which is part of the procedure validated for reading assessment in the Brazilian population (Cunha and Capellini, 2014). It is a 268 word-long narrative text that was extracted from the textbooks used in the public municipal city of Marilia (São Paulo, Brazil). The text was written on a sheet of paper in Times New Roman (size 12). The children were asked to read the text aloud as accurately and as fast as possible. They had to read the whole text, without interruption. Oral reading was recorded. The number of errors and time needed to read the whole text (transformed in wpm) were taken into account.

#### Phonological awareness

The Metalinguistic Skills Evaluation Battery (PROHFON Battery, Germano and Capellini, 2011, forthcoming), a standardized test, was used for the assessment of phonological skills. The test was designed for the Brazilian population of children from 8 to 13. In this test, pictures are used to suport processing and reduce phonological working memory load (Germano and Capellini, 2011, forthcoming). The three subtests of Phonemic Identification, Combination of syllables and combination of phonemes were administered, as processing similarly develops for the syllable and phoneme units in Portuguese (Duncan et al., 2013). Each subtest consisted in the presentation of five cards with six pictures each. The child was first asked to name the six pictures successively. When a picture was erroneously named, the experimenter provided the corresponding name. Then the child was asked to perform a phonological task and provide his/her response by pointing to one of the pictures. Each card was used for three successive trials, so that three pictures out of six had to be successively pointed at to provide the answer. For each task, the experiment began with three training trials (on one card of six pictures, only used for training), for which the child received corrective feedback. No feedback was provided during the experimental trials. There was no time pressure for providing the answer. Each correct pointing received one point, for a maximal score of 15 on each of the three tasks.

***Phoneme identification (PhoId).*** The experimenter said a single phoneme and the child had to point at the picture of the object whose name included the target phoneme. For example, the child was asked to point at the picture depicting the word that included the phoneme /b/ among “*balde*,” “*galinha*,” “*sapo*,” “*noz*,” “*pato*,” *and* “*cola*” (bucket, chicken, frog, nut, duck, glue). The task was designed to include one close distractor for each target phoneme (the /p/ of “*pato*” in the example). Three phonemes were successively provided so that three different pictures had to be successively pointed at on each card of six pictures. On each card, a single picture included each target phoneme. The child was instructed that the target phoneme could occur randomly at the beginning, in the middle or at the end of the phonological word.

***Syllable blending (SyBl).*** At each trial, two or three syllables were successively pronounced by the experimenter. The child was asked to blend them to form a word and then point to the corresponding picture. For example, a card was presented with the following pictures: “*sapo*,” “*chuva*,” “*coração*,” “*girafa*,” “*casa*,” and “*pé*” (frog, rain, heart, giraffe, house, and foot). The experimenter said “Take the first syllable of the word ‘cara’ /ka/ and blend it with the last syllable of the word ‘mesa’ /za/ which word do you get?” The child had to point to the picture of the word “*casa*,” among the six distractors. All target syllables were stressed syllables that could be located in initial, medial or final position within the phonological word.

***Phoneme blending (PhoBl).*** The instruction was very similar as for the syllable blending task except that two phonemes had to be blended to form a word. For example, the child was asked to blend the initial sound/phoneme of “*chapéu*” */∫/* with the last sound/phoneme of “mesa” /a/ and then point at the corresponding picture “chá.” The distractors for this trial were “*sol*,” “*pente*,” “*girafa*,” “*oca*,” and “*ovo*” (tea, sun, comb, giraffe, teepee, egg). “*Chá*,” “*ovo*,” and “*oca*” were target drawings for the three successive trials using the same set of six drawings.

#### Visual processing

Three different visual tasks were administered, two were taken from the test of visual perception skills (TVPS; Martin, 2006); the third one was the whole letter report task typically used to assess VA span abilities (Bosse et al., 2007).

The two subtests of visual closure (VC) and visual form constancy (VFC) of the TVPS (Martin, 2006) utilize black and white designs as stimuli and a forced choice paradigm. Each subtest starts with two training trials (not scored and with feedback) that are followed by 16 experimental items arranged in order of difficulty. The children can answer either verbally or by pointing at the target drawing. For each trial, a maximum of 20 s was provided for the response. Maximum score for each test is 16. Raw scores were recorded at the end of each subtest. The tests were applied individually, in a quiet room (without auditory or visual distraction).

***Visual form constancy subtest (VFC)***. A visual shape (the target) was presented at the top of a card. The child was asked to retrieve the top shape among five alternatives. The alternatives were either similar to the top shape while differing in size or orientation or were complex configurations, one of which included the target after transformation (rotation or size change). There was 1 practice trial and there were 16 test trials for a maximal score of 16. Each participant progressed on the subtest until he or she failed four out of five consecutive items.

***Visual closure subtest (VC).*** The black outline drawing of a complex geometrical shape was presented at the top of the sheet and the participant was asked to match it with one out of four fragmented shapes displayed below. The fragmented visual shapes of the distractors included similar features as the target but the orientation, the size or the position of some features had been changed. The target was a fragmented shape derived from the outline drawing. There was one training trial followed by 16 test trials.

***Letter global report task (LGR).*** On each trial, a five-consonant string was displayed for 200 ms at the center of the computer screen. The strings built-up from 10 consonants (BPTFLMDSRH) contained no repeated item. To prevent lexical activations, they never matched the skeleton of a real word. Each letter was used 10 times and appeared twice in each position. Letters were presented in upper case (Geneva 24) in black on a white background; each letter subtended a visual angle of 0.7°. To minimize crowding, the distance between adjacent letters was increased (inter-letter space of 0.57 cm). The whole array subtended an angle of ∼5.4°. The task began with 10 training trials, for which children received feedback. No feedback was provided on the experimental trials.

At the beginning of each trial, a central fixation point was presented for 1000 ms followed by a blank screen for 50 ms. Then, the consonant string was presented at the center of the display for 200 ms. Twenty experimental trials were displayed. The child had to report verbally (identification not location) the name of the letters he had identified. We measured the number of items accurately reported, irrespective of location, across the 20 experimental trials (maximum score = 100).

#### Procedure

The tests were applied in four different sessions in the following order: text reading first, then the PROHFON test of phonological awareness, then the two subtests of the TVPS and last the Global report task. The three phonological subtests were administered in a fixed order with phoneme identification first, then syllable combination then phoneme combination. The form constancy subtest of the TVPS was presented first followed by the VC test. The tests were administered individually in a quiet office at school. A short break was proposed between the different subtests.

#### Statistical analyses

We used *t*-tests (one tailed) to compare performance of the two groups (children with developmental dyslexia and typical readers on the reading), phonological awareness, and VA tasks. To explore the relationship between the different variables, a correlation analysis was computed on all the measures (a Bonferroni correction for multiple comparisons was applied) and for the whole population. Third, we carried out a principal components analysis (PCA) with *varimax* rotation on the data from the phonological and visual processing tasks to reduce the data set before exploring the concurrent predictors of reading fluency in the whole population. All factor loadings greater than or equal to 0.70 were used for interpretation. Finally, we examined the distribution of the individual phonological and visual factorial coefficients derived from PCA to identify those children with developmental dyslexia who exhibited a phonological or VA disorder. Children with developmental dyslexia whose phonological and visual factorial scores fell below the tenth percentile of the control group were considered as impaired on these factors.

## RESULTS

### OVERVIEW OF THE PARTICIPANTS’ PERFORMANCE

Performance of the control and children with developmental dyslexia on each experimental task is provided in **Table 2**. As a group, the children with developmental dyslexia took more than three times (31 versus 105 words per minute) as much time as the controls to read the whole text, thus showing a severe reading fluency disorder. Their performance was significantly lower than expected on the three tasks of phoneme identification, phoneme blending and syllable blending. They thus exhibited a phonological awareness disorder as typically reported in children with developmental dyslexia, regardless of language transparency. The children with developmental dyslexia further showed poor performance on the letter global report (LGR) task and the two subtests of the TVPS, thus showing a visual processing disorder in addition to their phonological disorder.

**Table 2:** Mean scores (and SD) of the control and children with developmental dyslexia for text reading, phonological awareness and visual processing.

Tasks	Developmental dyslexia group	Control group	*t* value, *p* value
**Text reading**			
Reading speed (wpm)	31,33 (15,57)	105,36 (26,08)	*t* = -14,00, *p* < 0.00001
Phonological awareness			
Phoneme identification (/15)	9,15 (3,37)	11,70 (2,68)	*t* = -3,39, *p* < 0.00001
Phoneme blending (/15)	5,87 (4,51)	13,84 (2,64)	*t* = -6,79, *p* < 0.00001
Syllable blending (/15)	8,70 (4,74)	13,84 (1,46)	*t* = -5,95, *p* < 0.00001
**Visual processing**			
Form constancy (/16)	5,15 (2,95)	10,45 (2,03)	*t* = -8,50, *p* < 0.00001
Visual closure (/16)	5,00 (3,13)	10,81 (2,43)	*t* = -8,43, *p* < 0.00001
Letter global report (/100)	50,88 (14,10)	65,21 (11,46)	*t* = -4,53, *p* < 0.00001

### RELATIONSHIP BETWEEN PHONOLOGICAL AWARENESS, VISUAL PROCESSING, AND READING

A correlation analysis was carried out on the measures of age, text reading, phonological awareness and visual processing for the whole population. When required data were log transformed to meet normality assumption. A Bonferroni correction was applied (significant at *p* < 0.0008). Results are reported on **Table 3**.

**Table 3:** Correlations among age, reading, phonological awareness, and visual processing for the whole population.

	CA	Reading	PhoI	PhoB	SyB	LGR	VC
Reading	-0.030 (*p* = 0.809)	–					
PhoI	0.163 (*p* = 0.189)	**0.427** (*p* < 0.0008)	–				
PhoB	-0.009 (*p* = 0.938)	**0.756** (*p* < 0.0008)	**0.570** (*p* < 0.0008)	–			
SyB	-0.083 (*p* = 0.508)	**0.664** (*p* < 0.0008)	**0.462** (*p* < 0.0008)	**0.789** (*p* < 0.0008)	–		
LGR	0.021 *P* = 0.864)	**0.456** (*p* < 0.0008)	0.166 (*p* = 0.184)	0.328 (*p* = 0.007)	0.355 (*p* = 0.003)	–	
VC	0.079 (*p* = 0.530)	**0.714** (*p* < 0.0008)	0.264 (*p* = 0.032)	**0.548** (p < 0.0008)	**0.494** (p < 0.0008)	0.403 (*p* = 0.001)	–
FC	-0.026 (*p* = 0.838)	**0.636** (*p* < 0.0008)	0.165 (*p* = 0.186)	**0.494** (*p* < 0.0008)	**0.426** (*p* < 0.0008)	**0.550** (*p* < 0.0008)	**0.622** (*p* < 0.0008)

*CA, chronological age; PhoI, phoneme identification; PhoB, phoneme blending; SyB, syllable blending; LGR, letter global report task; VC, visual closure subtest; FC, visual form constancy subtest. Bold: the correlations that remain significant after a Bonferroni adjustment (*p* < 0.0008).*

No correlation was found between chronological age and any of the reading, phonological or visual variables. In contrast, performance in text reading correlated with performance on both the phonological tasks (from 0.43 to 0.76) and the visual processing tasks (from 0.46 to 0.71). As expected, significant correlations were found between the three phonological tasks (from 0.46 to 0.79) and two out of the three visual processing tasks. Performances on the two subtests of VFC and VC correlate significantly. Global report performance does correlate with performance on Form constancy (0.55) but the correlation with the VC task is not significant after Bonferroni correction. As typically reported, performance in letter report does not correlate significantly with performance on any of the phonological tasks. In contrast, significant correlations were found between performance on the two subtests of VC and Figure Constancy and the two tasks of Phoneme and Syllable Combination. The overall data suggests that phonological and visual skills both relate to reading performance. The significant or close-to-significance relation between global report and the two subtests of the TVPS suggests that the three “visual” tasks share some common mechanisms. However, performance on VC and Form constancy but not Global report further relate to the two phonological tasks of phoneme and syllable blending suggesting that the former tasks probably tap additional cognitive processes not involved in global report.

### PREDICTORS OF READING PERFORMANCE

We computed a PCA with *varimax* rotation on performance for the three phonological tasks and the three visual processing tasks (**Table 4**). The analysis revealed a two-factor solution. The first factor (called the visual factor hereafter) accounted for 36.82% of the variance and received high loading from the shape constancy, VC and global report tasks. The second “phonological” factor that received high loadings from the three phonological tasks accounted for a further 36.28% of variance.

**Table 4:** Rotated factor loadings of the PCA for the visual and phonological subtests.

Tasks	Factor loadings	
	Factor 1: visual	Factor 2: phonological
Phoneme identification	-0,036	0,853*
Phoneme blending	0,398	0,829*
Syllable blending	0,393	0,770*
Shape constancy	0,866*	0,179
Visual closure	0,720*	0,361
Letter global report	0,789*	0,070

*p < 0.001.

The individual factorial coefficients were then used to explore the concurrent predictors of text reading performance in the whole population of children with developmental dyslexia and control participants. Two hierarchical regression analyses were computed for which the two factors of interest were entered alternately at step one or step 2 to assess their unique contribution to text reading performance. As age did not correlate with reading performance, this variable was not entered in the analysis. Results of the regression analyses showed that the visual factor accounted for 37.7% of unique variance in text reading fluency and the phonological factor for a further and independent 33.8% of variance.

### IDENTIFICATION OF COGNITIVELY DISTINCT SUBTYPES OF CHILDREN WITH DEVELOPMENTAL DYSLEXIA

The regression analyses have shown that the phonological and “visual” factors contribute independently to text reading performance in our group of Portuguese-speaking children with developmental dyslexia and control participants. We then explored whether different cognitive subgroups could be identified in our population of children with developmental dyslexia based on their phonological and visual factor coefficients. The children with developmental dyslexia with a coefficient below the tenth percentile on one or the other factor (cut-off at -0.13 and -0.56 for the visual and phonological factors respectively) were considered as showing a cognitive disorder.

**FIGURE 1:**
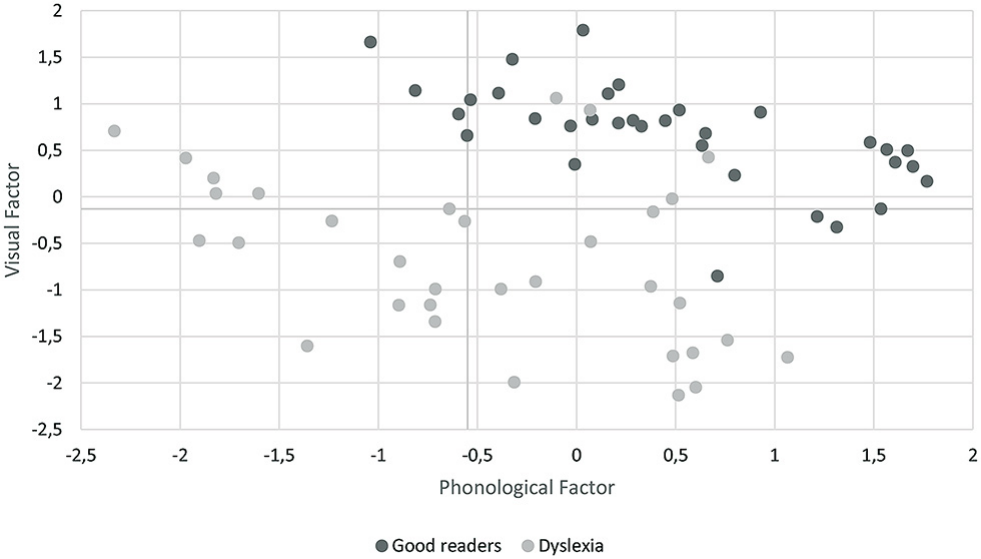
**Scatterplot’s graphic of the distribution of children with developmental dyslexia and control group based on their phonological and visual coefficients**.

**Figure 1** shows the scatterplot of the participants based on their phonological and visual coefficients. Unexpectedly only five children (15.1%) showed poor phonological skills but normal visual processing, thus defining a group with a selective phonological disorder. In contrast, a high proportion of children (72.7%) exhibited a visual processing disorder. This group of children split into a first subgroup of 13 children (39.4%) who showed a selective visual disorder (in the absence of phonological problems) and a second subset of 11 children (33.3%) with both visual and phonological disorders. Only four children with developmental dyslexia (12.1%) showed no phonological or visual disorder.

## DISCUSSION

The results can be summarized as follows. First, Brazilian Portuguese children with developmental dyslexia show significant deficits not only in phonological processing but further in visual processing tasks that require multi-element visual processing. Second, each of these phonological and visual processing abilities account for a substantial amount of unique variance in reading fluency. Last, different cognitively homogeneous subtypes can be identified in the Brazilian Portuguese population of children with developmental dyslexia, in particular two subsets of children who show either a single phonological disorder or a single visual processing disorder. Thus the current findings extend previous data from more opaque orthographies as French and English, in showing the importance of investigating visual processing skills in addition to phonological skills in children with developmental dyslexia whatever their language orthography transparency.

### VISUAL PROCESSING DISORDERS IN BRAZILIAN PORTUGUESE CHILDREN WITH DEVELOPMENTAL DYSLEXIA

One of the main finding of the current study is to show that Brazilian Portuguese children with developmental dyslexia exhibit a visual processing disorder and more generally that visual processing abilities contribute to reading performance in Brazilian Portuguese children, independently of their phonological skills. In the current study, participants were administered three different visual processing tasks: a LGR task known to reflect VA span abilities (Bosse et al., 2007) and two tasks of VC and visual constancy taken from the TVPS (Martin, 2006). Results showed substantial correlations between these three visual tasks and strong correlations between these tasks and reading fluency. Hierarchical regressions revealed that the visual factor accounted for 38% of unique variance in reading fluency, suggesting a substantial and independent contribution of visual processing skills to reading performance in Brazilian Portuguese. These findings are consistent with a number of previous data that emphasized the importance of visual processing in reading acquisition and developmental dyslexia (Bosse et al., 2007; Vidyasagar and Pammer, 2010; Franceschini et al., 2012). However, children with developmental dyslexia do not have a general visual processing disorder (Shovman and Ahissar, 2006). Recent evidence suggests that children with developmental dyslexia may suffer from VA deficits rather than low-level perceptual disorders (Boden and Giaschi, 2007). A series of studies demonstrated visual spatial attention orienting disorders and sluggish attentional shifting in developmental dyslexia (Facoetti and Molteni, 2001; Hari and Renvall, 2001). However, these disorders extended to the auditory modality (Gaab et al., 2007; Lallier et al., 2009; Facoetti et al., 2010) and were typically found to be associated with phonological problems (Facoetti et al., 2006; Lallier et al., 2013). In contrast, there is strong evidence that VA span disorders, as assessed through global and partial letter report tasks, typically dissociate from phonological problems in the population of children with developmental dyslexia. Evidence initially came from behavioral studies on French and English speaking children (Bosse et al., 2007). It was shown first that French and English speaking children with developmental dyslexia performed worse on the VA span tasks than chronological age matched controls; second that VA span abilities in the normal and children with developmental dyslexia taught in languages with opaque orthographies contributed to their reading performance (accuracy and speed) independently of the children’s phonological skills. The current study extends these findings in showing that VA span disorders are associated with developmental dyslexia in Brazilian Portuguese, a more transparent orthography than French or English. As previously described in more opaque languages, correlation analyses in the current study further showed that performance on the LGR task does not correlate with performance on phonological skills, thus suggesting that these tasks tap independent cognitive mechanisms.

The use of letter strings and oral report to assess VA span abilities was criticized arguing that the poor performance of children with developmental dyslexia in letter report tasks may reflect visual-to-phonological code mapping difficulties rather than a visual multi-element parallel processing disorder (Ziegler et al., 2010). A series of studies were carried out to support the visual attentional interpretation of the VA span disorder in developmental dyslexia (Lassus-Sangosse et al., 2008; Valdois et al., 2012). Critical arguments were derived from behavioral studies showing that VA span impaired children with developmental dyslexia were similarly impaired in non-verbal tasks requiring multiple non-verbal stimuli processing (Lobier et al., 2012a,b) and from neuroimaging studies showing that the VA span disorder related to underactivation of visual-attention-related parietal regions (namely the superior parietal lobules bilaterally; Peyrin et al., 2011, 2012; Reilhac et al., 2013). In line with these findings, the current study demonstrates significant relationships between the LGR task and the two non-verbal tasks of VC and form constancy. Evidence that the three tasks contribute to a “visual” factor that independently correlates to reading performance cannot be easily reconciled with a phonological interpretation of the VA span disorder. These findings rather suggest that these two non-verbal matching tasks share some common visual mechanism with the LGR task and reading performance. Although the VC and visual constancy task are not “pure” VA tasks, these two tasks and the letter report task might require allocating VA across distinct visual elements to form an integrated perceptual representation of the whole stimulus.

It is well admitted that perceptual completion arises from processing of a stimulus as a whole (Boucard and Humphreys, 1992). At the behavioral level, performance in perceptual completion was found to correlate with performance in visual search, thus suggesting that both tasks may share common VA processes (Amson and Johnson, 2006). Evidence that visual search performance further relates to letter report tasks (Lallier et al., 2009, 2013) suggests that perceptual completion performance may involve similar VA processes. The hypothesis that VA could mediate the VC – letter report task relationship also find some support at the neurobiological level as the two tasks involve similar parietal regions (Schlesinger et al., 2013; Moratti et al., 2014).

The VFC subtest was reported as correlating with the VC Subtest (Martin, 2006). The visual constancy task requires forming a mental representation of the target shape as a whole and mentally transforming this global shape to match one of the alternatives. Like VC and VA span tasks, parietal regions including the superior parietal lobes were found activated while children performed mental transformations (Jordan et al., 2001; Cohen et al., 2008). We provide here evidence that performance in visual constancy relate to reading performance as previously reported by Siok and Fletcher (2001). Evidence for a form constancy and VC disorder has been previously reported in Chinese children with developmental dyslexia (Ho et al., 2002) as for the Brazilian Portuguese children in the current study. The overall findings clearly show that visual (attentional) processing abilities contribute to reading performance in Brazilian Portuguese children with developmental dyslexia and typically developing children.

### COGNITIVELY DISTINCT SUBTYPES OF DEVELOPMENTAL DYSLEXIA IN THE BRAZILIAN POPULATION

The second main finding of the current study is to show that phonological processing skills and visual processing abilities contribute independently to reading fluency in the Brazilian Portuguese population and that each cognitive disorder defines specific subtypes of developmental dyslexia. It has already been shown that phonological awareness plays a key role in reading acquisition and developmental dyslexia across alphabetic orthographies (Byrne, 1998; Landerl et al., 2013). Accordingly, phonological skills were found impaired in our Brazilian Portuguese children with developmental dyslexia participants and phonological skills explained 33% of unique variance in reading fluency for the whole population. Several studies in transparent orthographies have reported a weaker influence of phonological awareness on reading fluency. Even in opaque languages, phonological awareness more strongly relates to reading accuracy than reading fluency (Moll et al., 2013). Moreover in transparent orthographies, the predictive strength of phonological awareness typically decreases after only a few years of literacy instruction (1 or 2 years) so that the impact of phonological awareness is weaker (Vaessen et al., 2010). This may partly account for the moderate contribution of phonological skills to reading fluency in the current study. Interestingly, the same phonological skills and visual abilities reported as related to reading performance in opaque languages were found to contribute to reading fluency in a more transparent orthography as Brazilian Portuguese. This suggests that languages that vary in orthographic depth nevertheless recruit similar cognitive processes.

More importantly, the results from our sample of Brazilian Portuguese children with developmental dyslexia replicate previous findings from more opaque orthographies (French and English). In all three languages, separate subgroups of children with developmental dyslexia were identified as having a single phonological disorder, a single visual disorder, the two disorders or none of them. Moreover in Brazilian Portuguese as in more opaque orthographies, a majority of the children with developmental dyslexia was found to exhibit a single cognitive disorder, either phonological (15%) or visual (39%). Although the involvement of common VA mechanisms in the LGR tasks and the two tasks of VC and visual constancy remains to be fully demonstrated, the overall findings suggest that phonological and VA disorders may independently contribute to the poor reading outcome of children with developmental dyslexia, whatever the orthographic transparency in which they are taught. Future studies are needed to investigate more deeply the relationship between letter report VA span tasks and tasks of VC or visual constancy. Additional studies should explore VA span abilities in children with developmental dyslexia from transparent orthographies to confirm the importance of multi-element parallel processing in developmental dyslexia.

Last, the current findings highlight the heterogeneity of the Brazilian Portuguese population with developmental dyslexia, suggesting that no one explicit remedial instructional program can successfully address the needs of every child. Intensive remediation programs focused on children with developmental dyslexia’s underlying phonological disorders have proven useful to improve their decoding skills (Ehri et al., 2001; Simos et al., 2002; Griffiths and Stuart, 2013). However, positive effects of such programs do not always generalize to fluent text reading and some children with developmental dyslexia are resistant to phonology-based remediation (Shaywitz et al., 2008). Investigation of our Brazilian Portuguese sample of children with developmental dyslexia revealed that a non-trivial number of children in this population exhibits no phonological disorder but a VA deficit. Recent evidence suggests that selective VA span remediation programs are successful to improve reading performance (accuracy and fluency) in children with an underlying VA span disorder but such positive effects may be more sensitive in opaque than transparent orthographies (Valdois et al., 2014). Investigation of the effect of such VA span remediation programs on reading fluency in a group of Brazilian Portuguese children with developmental dyslexia with visual disorders but no phonological problems would help better understanding the origin of their reading difficulties.

## Conflict of Interest Statement

The authors declare that the research was conducted in the absence of any commercial or financial relationships that could be construed as a potential conflict of interest.
